# 

**Published:** 1887-12-10

**Authors:** 


### QUESTIONS AND ANSWERS.

### WITHOUT HOSPITAL ACCOMMODATION?

Mr. Robert Davidson, Hon. Sec. Tynemouth Infirmary, asks:—"Is there a town or borough in the United Kingdom of 50,000 inhabitants without an hospital or infirmary other than the workhouse hospital for paupers, or the hospital for infectious diseases belonging the Corporation?"

⁂ After careful inquiry we are satisfied that it may be safely asserted that there is no town or borough in the United Kingdom having a population of 50,000 inhabitants which is entirely destitute of hospital accommodation.—Ed. T. H.

The letter of "A Private Nurse" is too long for publication.

**NOTICE TO CORRESPONDENTS.**

All correspondence must be written on one side of the paper only.

We cannot undertake to return MSS. not used.

**Communications,** whether intended for insertion or for private information, must be authenticated by the names and addresses of the writers, not necessarily for publication.

**Local papers** containing reports or news paragraphs **should be marked.**

It is especially requested that early intelligence of **local events of interest,** or which it may be thought desirable to bring under notice, may be sent direct to the Editor.

**All communications respecting editorial matters** should be addressed to the Editor, Norfolk House, Norfolk Street, Strand, London, W.C.

### Amusements with Profit for all Ages.

**Rules.**—All answers must be addressed to the Prize Editor, The Hospital, Norfolk House, Norfolk Street, Strand, W.C., not later than the *first* post on Thursday next. The full name and address must in all cases be given, but a *nom de plume* may be added for publication. Only one side of the paper must be written on.

### FOR MEN AND WOMEN.

### Work Competition.

I. A PRIZE of THREE GUINEAS will be given to the competitor who sends in the best specimen of painting, which may take the form of a picture, panel, screen, flower-pots, etc., or illuminations.

II. TWO GUINEAS will be given for the best dressed doll or other piece of work suitable for presentation to the inmates of the hospitals.

III. ONE GUINEA will be given for the best scrap-book.

### Acrostic Competition.

A PRIZE of ONE GUINEA will be given to the competitor who gains the highest number of marks for best original acrostics during the ensuing quarter. All acrostics sent in for this competition will be credited according to merit, twelve marks being the highest score given for one acrostic.

A PRIZE of HALF-A-GUINEA to be given to the competitor who gains the highest score for solution of acrostics set, including the one inserted on November 26th. One mark only will be given to the competitors who solve all the lights of each acrostic.

These competitions to alternate weekly.

*Next week*, therefore, credit will be given for the *best original acrostic.*

No. 2.—Double Acrostic.

Pleasant rest for weary feet,
To thirsty ears a music sweet.

1. Ride on me when afield you go,
Or in me when your funds are low;
But don't cut meat in pieces so.

2. Precursor to a festival,
Our Mother by this name we call;
When day towards its close doth fall.

3. Do me rightly every day,
One part only of the play;
You do deliver this, you say.

4. I claim it from thee,
But 'tis not left to me;
We should do it, you see.

5. Patient labour on the soil,
Space of time to wait awhile;
When it is full the owners smile.

6. Bring it quickly by main force,
Healthy looks, but not too coarse;
Of legal wisdom one great source.

### Answers.

No. 1—Double Acrostic.

A rie L
U ndin E
T iar A
U — V
M elpomen E
N imbu S

Correct answers have been received from Boss, Canice, Dunce, Ellice, Gretta, Condorcet, Brownie, K. M. Ostrich, Nogrub, Toby, Mater, and Tabs.

### II.—Word and Literary Competition.

Commenced November 5th, 1887; ends January 28th, 1888.

Three prizes of one guinea, 15*s*. and 10*s*. 6*d*. will be given each quarter to the three competitors who obtain the highest number of marks: (1) by making the largest number of words derived from the word or phrase set for anatomical dissection; or (2) for the best answers to (6); or (3) by (1) and (2) combined.

(A) THE WORD COMPETITION.

We shall give the newspapers for dissection during this quarter, that for this week being "Morning Post."

Observe.—Proper names, abbreviations, foreign words, words of less than four letters and repetitions are barred. Nuttall's *Standard* dictionary only to be used.

### Results of Fourth Week.

*Accepted Scores*—Ellice (32), Gretta (29), Tim (27), Reldas (25), E. E. (23), Aralc 22), Skye (22), Brownie (22), Kath (21), K. M. (20), Puss (18), Lauriston (17), Condorcet (16), Toby (16), Nogrub (15), Ostrich (15), Brisbane (14).

(B) THE LITERARY COMPETITION.

For particulars of this see page 157, Nov. 26th. *Ellice* sends us a letter from Tauranga, N.Z *Fred* one from Cambridge Gulf. We should be glad of further contributions.

### THE THEATRES.

Miss Mary Anderson must be congratulated on having met with more success in the *Winter's Tale* at the Lyceum than in any other character she has yet represented in London. It would be vain to attempt to find a true reason for this success in the excellence of her acting, as it would be too much to say that she ever excites an enthusiasm amongst her auditors; but most are pleased, and for the most part recommend their friends to follow their example, and go and see the latest Shakespearian revival. We have always looked upon the *Winter's Tale* as one of the least entertaining of Shakespeare's plays for study, and one of the least likely to prove a draw on the stage. As the present revival has proved an unqualified success, all the more credit is due to the management of the Lyceum for the excellent manner in which the play is presented. The plot is generally well known; but as it may not be familiar to some of our readers, it will be well to give a short outline. It is a comedy in five acts, and between acts three and four fifteen years are supposed to elapse. The interest in each part centres in a different individual; Hermione being the heroine of the first part, Perdita (her daughter) of the second. At the opening of the play Leontes, king of Sicily, is entertaining Polixenes, king of Bohemia, the old playfellow of his youth, and, perceiving the latter's unconcealed admiration of Hermione, conceives the most violent jealousy of his guest, and plots against his life. Polixenes realizes that he has outstayed his welcome, and cuts short his visit at the advice of Camillo, who has been appointed his cup-bearer. His sudden departure is taken by Leontes to be but a further proof of his wife's guilt, and Hermione is arraigned before the high court of Sicily. In spite of her able pleading and the declaration of Apollo that "Hermione is chaste, Polixenes blameless, Leontes a jealous tyrant," she is condemned without mercy, and, falling in a swoon, is carried from the stage senseless. Act IV. is laid in Bohemia, and its chief interest lies in the love scene between Florizel, the king's son and heir, and Perdita the supposed daughter of a lowborn shepherd. The young prince refuses to obey the orders of his father to give up the girl, and, fleeing with her to the Court of Leontes, is well received. It is there discovered that Perdita is really the lost child of Leontes, whom he had endeavoured to destroy in his jealous rage, and is willingly given to Florizel. It is found further that Hermione is not really dead, and the play concludes with a most effective tableau, in which the King is shown a statue, as he believes, but which turns out to be his wife, Hermione. The great success of the play is certainly to be attributed more to the magnificent mounting, scenery, and dresses which are used in this picturesque representation, than to the personal merits of actors and actresses; though such success would never be obtained by an incapable company. Miss Anderson takes upon herself the double parts of Hermione and Perdita. She does equally well in both parts, but never rises to the height of a great actress. She is painstaking throughout, and always bears a most attractive presence on the stage, but never impresses her audience, and there is no danger of their forgetting the woman in the character she is assuming. She has a great opportunity in the speech to the Court, but she does not grasp it. Her voice is harsh; her demeanour is studied, but lacks passion; we hear everything we feel nothing. As Perdita she looks charming, and with Mr. Fuller Mellish takes part in a most effective dance—the only part of her performance which calls for that genuine applause which theatre habitué know how to appreciate at its true value. In the last tableau her representation of the statue is very effective. Mr. Forbes Robertson takes the part of Laertes, the principal male character, with considerable success. He does well without making a mark, though his performance is far more feeling and artistic, as contrasted with cultured, than the representation of Miss Anderson. The minor parts are filled by an efficient company, of whom Mr. Chas. Collette shines forth pre-eminent as Autolycus, the rogue: small as his part is, and merely incidental to the plot of the story, he is greeted with the most hearty applause, and does much to relieve the play of the stiffness from which it might otherwise suffer. No one could ever become enthusiastic over this play, but it has a genuine hold on the public which it deserves in many respects, and is certainly one of the most pretty and complete shows ever presented even at the Lyceum.

### THE CHILDREN'S CORNER.

PRIZES.

FOR MOST CORRECT ANSWERS TO PUZZLES.

This Commenced October 1st, 1887.

One Pound a Month.

FOR BEST LITTLE LETTERS.
Ten Shillings per Quarter.

A PRIZE OF THREE GUINEAS

to the Competitor who shall have sent in the largest number of correct or best answers during the twelve months.

### Puzzles—Second Week.

No. 5. Enigma.

My first represents numbers,
My second magnifies numbers,
My third adds to numbers,
My whole doubts numbers.

No. 6. Diamond Puzzle.—1. A consonant. 2. Not near. 3. A favourite pastime. 4. A seaside town. 5. Birds. 6. A tool. 7. A consonant.

No. 7. Towns in England Anagrammatically Expressed. 1. He sold us this. 2. A mutton shop. 3. We've hit a hen. 4. Mangle not I.

No. 8. Arithmetical Puzzle.—Two boys had been buying peaches, when one of them said to the other, "Give me one of your peaches, and I will have as many as you." "No!" said the other; "but give me one of yours, and I will have double the number you have." How many had each?

### Results of Fourth Week Puzzles.

No. 13 Double Acrostic.

L ouvier S
Y enise I
O rwel L
N asi K
S amo S

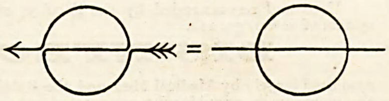

No. 14. The arrow in the above diagram shows the way the pen should go, several competitors have crossed the line twice.

No. 15. Biographical Charade —Gold-Smith = Goldsmith.

No. 16. Enigma.—Mankind.

*All right*—M. S. W., The Young Canadian, A. C. K. *Three right*—Judy, Mount Edgecumbe, Poodle, Jumps, Gem. *Two right*—Pepita, Paddy. *One right*—Skye, Ossian, Fern, Alsie, W. R. Mills. A. C. K.—In error you were only credited with three marks on Nov. 19th.

### Results of Second Monthly Competition.

The competition has been closer than ever. A. C. K. and M. S. W. have succeeded in gaining the highest score (16 marks), but as A. C. K gained half of last month's prize, he is, according to the rule, not entitled to take any of this; therefore M. S. W. (Master M. S. Williams, 16, Shelley Road, Worthing) takes the whole. Poodle and Pepita have only lost their chance by two marks. I hope, therefore, that they and all other competitors will work doubly hard to come in first at the end of this month.

### Letters.

Next week tell me how you would like to spend "Christmas Day."

**Notice to all Correspondents.**—N.B.—All letters referring to this page, which do not arrive at Norfolk House, Norfolk Street, Strand, W.C., by the *first post on Thursdays*, and are not addressed PRIZE EDITOR, will in future be disqualified and disregarded.

No one will be permitted to win the Monthly or Quarterly Prizes twice in any one year, the annual prizes being offered especially to prevent this.

